# Oestrogen receptor β regulates epigenetic patterns at specific genomic loci through interaction with thymine DNA glycosylase

**DOI:** 10.1186/s13072-016-0055-7

**Published:** 2016-02-16

**Authors:** Yun Liu, William Duong, Claudia Krawczyk, Nancy Bretschneider, Gábor Borbély, Mukesh Varshney, Christian Zinser, Primo Schär, Joëlle Rüegg

**Affiliations:** Department of Biochemistry and Molecular Biology, Key Laboratory of Metabolism and Molecular Medicine, Ministry of Education, Fudan University Shanghai Medical College, Shanghai, People’s Republic of China; Department of Biomedicine, University of Basel, Mattenstrasse 28, 4058 Basel, Switzerland; Genomatix Software GmbH, Bayerstr, 85a, 80335 Munich, Germany; Swedish Toxicology Science Research Center (Swetox), Forskargatan 20, 151 36 Södertälje, Sweden; Department of Clinical Neurosciences, Karolinska Institutet, CMM L8:00, 171 76 Stockholm, Sweden; Department of Biosciences and Nutrition, Karolinska Institutet at Novum, 141 83 Stockholm, Sweden; Novartis Institutes for BioMedical Research, Novartis Pharma AG, Werk Klybeck, 4002 Basel, Switzerland

**Keywords:** Oestrogen receptor β, Thymine DNA glycosylase, DNA methylation, Reduced representation bisulfite sequencing, Mouse embryonic fibroblasts, Mouse embryonic stem cells

## Abstract

**Background:**

DNA methylation is one way to encode epigenetic information and plays a crucial role in regulating gene expression during embryonic development. DNA methylation marks are established by the DNA methyltransferases and, recently, a mechanism for active DNA demethylation has emerged involving the ten-eleven translocator proteins and thymine DNA glycosylase (TDG). However, so far it is not clear how these enzymes are recruited to, and regulate DNA methylation at, specific genomic loci. A number of studies imply that sequence-specific transcription factors are involved in targeting DNA methylation and demethylation processes. Oestrogen receptor beta (ERβ) is a ligand-inducible transcription factor regulating gene expression in response to the female sex hormone oestrogen. Previously, we found that ERβ deficiency results in changes in DNA methylation patterns at two gene promoters, implicating an involvement of ERβ in DNA methylation. In this study, we set out to explore this involvement on a genome-wide level, and to investigate the underlying mechanisms of this function.

**Results:**

Using reduced representation bisulfite sequencing, we compared genome-wide DNA methylation in mouse embryonic fibroblasts derived from wildtype and ERβ knock-out mice, and identified around 8000 differentially methylated positions (DMPs). Validation and further characterisation of selected DMPs showed that differences in methylation correlated with changes in expression of the nearest gene. Additionally, re-introduction of ERβ into the knock-out cells could reverse hypermethylation and reactivate expression of some of the genes. We also show that ERβ is recruited to regions around hypermethylated DMPs. Finally, we demonstrate here that ERβ interacts with TDG and that TDG binds ERβ-dependently to hypermethylated DMPs.

**Conclusion:**

We provide evidence that ERβ plays a role in regulating DNA methylation at specific genomic loci, likely as the result of its interaction with TDG at these regions. Our findings imply a novel function of ERβ, beyond direct transcriptional control, in regulating DNA methylation at target genes. Further, they shed light on the question how DNA methylation is regulated at specific genomic loci by supporting a concept in which sequence-specific transcription factors can target factors that regulate DNA methylation patterns.

**Electronic supplementary material:**

The online version of this article (doi:10.1186/s13072-016-0055-7) contains supplementary material, which is available to authorised users.

## Background

DNA methylation and histone modifications are ways to encode epigenetic information and play a crucial role in regulating gene expression during embryonic development [1, 2]. Aberrant epigenetic patterns are found in various human diseases, including cancer, obesity, and psychiatric disorders [3].

The most common epigenetic DNA modification is methylation at the fifth position of cytosine (5mC). The DNA methylation pattern is established and maintained by DNA methyltransferases (DNMTs) that transfer methyl groups from S-adenosyl methionine to cytosines, mainly at CpG dinucleotides [4]. DNA methylation has long been considered as fairly stable and only removable by passive mechanisms, i.e. by inhibition of DNMT activity during DNA replication. More recently, however, pathways of active DNA demethylation have been found to operate during embryonic development, primordial germ cell maturation [5], and cell differentiation [6]. Active demethylation is initiated by the oxidation of 5mC to 5-hydroxymethylcytosine (5hmC) by ten-eleven translocation (TET) proteins, a family of Fe(II)- and 2-oxoglutarate-dependent DNA dioxygenases [7]. Genome-wide mapping revealed that 5hmC is mostly found in pluripotent cells and neurons, in bodies of transcribed genes, and in gene regulatory regions (promoters and transcriptional enhancers) [8], often concomitant with the bivalent chromatin marks lysine 4 di- and tri-methylation and lysine 27 tri-methylation at histone H3 (H3K4m2/3 and H3K27m3, respectively) [9]. Such regions are poised for activation or permanent silencing during lineage commitment and terminal cell differentiation [10]. 5hmC can be further processed to 5-formylcytosine (5fC) and 5-carboxylcytosine (5caC) by TET proteins. These modifications are recognised and excised by the thymine DNA glycosylase (TDG) [11] and replaced by an unmethylated cytosine by the base excision repair [12]. Evidence for active DNA demethylation by this mechanism stems from the findings that *Tdg* deficiency is embryonic lethal in mice [13, 14] and leads to changes in the distribution of cytosine modifications during stem cell differentiation [13, 15, 16], in particular in gene regulatory regions such as promoters and enhancers. Further, 5fC and 5caC accumulate in the absence of *Tdg* in embryonic stem cells (ESCs) at promoter and enhancer regions [15, 16].

An open question is how factors involved in regulation of DNA modifications are targeted to specific genomic loci. It has been suggested that transcription factor binding to their recognition sites leads to de novo methylation at proximal regions [17–19]. Further, non-coding RNAs are thought to guide DNMTs [20–22] or enzymes involved in active DNA demethylation [23] to specific regions, resulting in silencing or activation of these loci, respectively. Nevertheless, the exact mechanism of how DNA methylation is regulated at specific genomic regions is still not well understood.

Nuclear receptors (NRs) are inducible transcription factors that have been suggested to regulate epigenetic events, particularly histone modifications [24] but also DNA methylation [25–30]. Previously, we reported that the NR oestrogen receptor beta (ERβ) protects a single CpG in the promoter region of glucose transporter 4 (*Glut4*) from being hypermethylated [25]. Hypermethylation of this CpG in the absence of *ERβ* correlated with changes in expression and inducibility of *Glut4*. Thus, ERβ shows features of a transcription factor involved in the local regulation of DNA methylation.

ERβ is one of the two ER isoforms that mediate the physiological effects of oestrogens, the female sex hormones. It is involved in the development and functioning of the reproductive organs, but also of other tissues, e.g. the brain [31] and adipose tissue [32]. It is mostly found in the cell nucleus where it, upon activation, binds to regulatory elements [oestrogen response elements (EREs)] at target genes. There are a number of co-activators that enhance ER transcriptional activity, including chromatin-remodelling factors [33]. The ERs are not only activated by endogenous hormones, but also by pharmaceuticals and food-derived compounds such as phytoestrogens, plant protection products, and plasticisers. Exposure to a number of these compounds was shown to induce alterations of DNA methylation [34, 35].

Based on our previous results, we set out to analyse the effects of ERβ deficiency on DNA methylation at a genome-wide level. Using reduced representation bisulfite sequencing (RRBS) in mouse embryonic fibroblasts (MEFs) derived from wildtype (wt) and ERβ knock-out mice, we identified more than 8000 differentially methylated positions (DMPs). Differences in methylation correlated with changes in expression of the nearest genes and were reversible by re-introducing ERβ into knock-out MEFs. Further, we show here that ERβ interacts with TDG and that TDG is ERβ-dependently recruited to identified DMPs. Thus, we provide evidence that ERβ plays a role in regulating DNA methylation at specific genomic loci by targeting TDG to these regions.

## Results

### ERβ deficiency leads to methylation changes in developmental genes

**Fig. 1:**
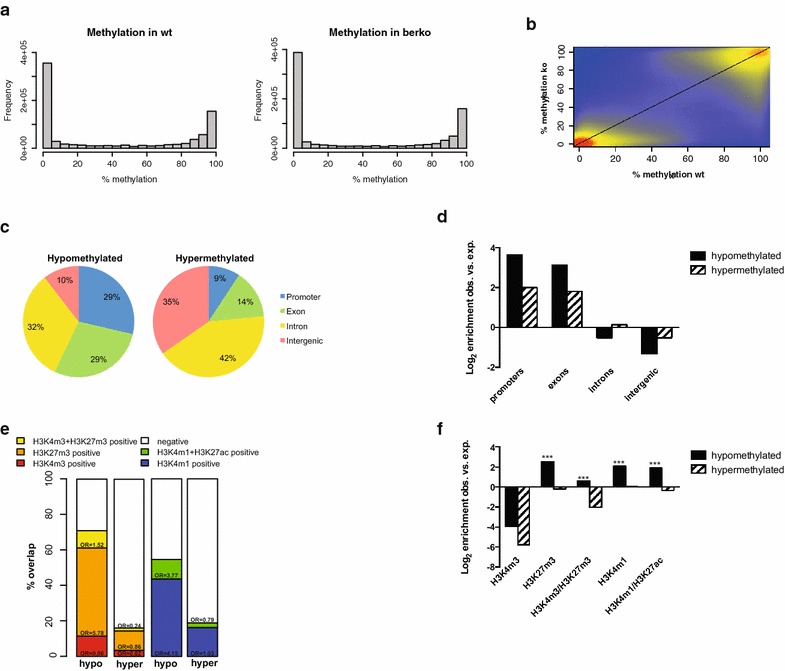
ERβ deficiency leads to altered DNA methylation patterns. **a** Histogram showing the distribution of methylation at the sequenced cytosines in wt and βerko MEFs. **b** Scatterplot of percentage (%) methylation in wt vs. βerko MEFs at cytosines covered in both cell types. **c** *Pie chart* presenting the genomic distribution of hypo- and hypermethylated positions. A position was considered hypermethylated if more than 80 % of the reads indicated methylation and hypomethylated if less than 20 % indicated methylation in βerko MEFs. **d** Enrichment (log2 ratios of observed over random) of hypo- and hypermethylated positions at different genomic features. **e** Comparison of regions identified by RRBS with datasets for histone modifications in MEFs [36] using GenomeInspector (Genomatix). *Bar plots* indicate percentages of hypomethylated (hypo) and hypermethylated (hyper) CpGs either marked by H3K4m3 (*red*), H3K27me3 (*orange*), both marks (*yellow*), or none of them (negative, *white*), or H3K4m1 (*blue*), H3K4m1 plus H3K27ac (*green*), or none of them (negative, *white*). Odds ratios (ORs) and *p* values according to Fisher exact test. **f** Enrichment (log2 ratios of observed over random) of histone modifications at hypo- and hypermethylated positions

To identify genomic loci that show DNA methylation changes in the absence of ERβ [GenBank: NM_207707, Swiss-Prot: O08537], we conducted reduced representation bisulfite sequencing (RRBS [36]) with MEFs derived from *ERβ*^+*/*+^ (wt) and *ERβ*^−*/*−^ (βerko) mice [25]. Sequencing resulted in roughly 40 million reads of which 40 % were unambiguously mapped to the mouse genome. Around 3 × 10^5^ CpGs were covered by the screen, which corresponds to 2.5 % of all CpGs in the mouse genome. In both cell lines, around 52 % of the covered CpGs were unmethylated (<20 % methylation), around 33 % fully methylated (>80 % methylation), and around 15 % displayed methylation between 20 and 80 % (Fig. 1a). The majority of positions were fully methylated or unmethylated in both cell types (Fig. 1b). We chose to focus on CpGs which were covered by more than four reads in both cell types and were either unmethylated (<20 % reads indicating methylation) in wt and methylated (>80 % methylation) in βerko cells or vice versa. These criteria identified 8071 DMPs, 6016 of which were highly methylated in wt and less methylated in βerko MEFs (hereafter referred to as hypomethylated DMPs) and 2055 were highly methylated in βerko and less methylated in wt MEFs (hereafter referred to as hypermethylated DMPs). Two thousand nine hundred and fifteen hypo- and 133 hypermethylated CpGs were found in clusters, forming 466 and 32 clusters, respectively. Annotation of DMPs showed the expected enrichment for promoter and intragenic regions compared to the whole genome (Fig. 1c, d; Table 1). However, gene-associated regions were more often found in hypo- than in hypermethylated loci (Table 1). Gene ontology (GO) analysis of genes containing DMPs showed enrichment for pathways involved in developmental processes for both hypo- and hypermethylated genes (Table 2).

**Table 1:** Genomic distribution of hyper- and hypomethylated differentially methylated positions (DMPs)

	% in genome	% in hypermethylated	Enrichment in hypermethylated	% in hypomethylated	Enrichment in hypomethylated
Exonic	5.5	19.1	3.5	48	8.7
Intronic	37.4	38.2	1	27	0.7
Intergenic	57.1	42.6	0.7	25	0.4
Promoter	2.6	10	3.8	30.8	11.8

**Table 2:** Gene ontology (GO) enrichment analysis of genes whose promoter is either hyper- or hypomethylated

GO term	*p* value	# Genes (63 in total)
Hypermethylated genes		
Embryonic morphogenesis	4.91E−04	5
Embryonic appendage morphogenesis	6.39E−04	3
Embryonic limb morphogenesis	6.39E−04	3
Regulation of transcription, DNA dependent	7.62E−04	8
Regulation of RNA metabolic process	8.50E−04	8
Transcription, DNA dependent	1.01E−03	8
RNA biosynthetic process	1.03E−03	8
Appendage morphogenesis	1.12E−03	3
Limb morphogenesis	1.12E−03	3
Embryonic development	1.19E−03	6

GO term	*p* value	# Genes (223 in total)
Hypomethylated genes		
Developmental process	5.29E−10	52
Multicellular organismal development	2.70E−09	48
Anatomical structure development	4.73E−09	44
System development	1.93E−08	41
Regulation of transcription from RNA polymerase II promoter	6.70E−08	20
Regulation of transcription, DNA dependent	7.84E−08	27
Regulation of RNA metabolic process	1.10E−07	27
Positive regulation of cellular biosynthetic process	1.42E−07	20
Transcription from RNA polymerase II promoter	1.66E−07	20
Transcription, DNA dependent	1.88E−07	27

Included genes show more than 30 % difference in methylation between wt and βerko MEFs, and at least 30 % of all promoter CpGs are covered by more than four reads in both cell types

### Hypomethylated regions overlap with silenced transcriptional regulators

To further characterise the genomic regions containing DMPs, we compared our data obtained by RRBS with published datasets for enrichment of different chromatin marks. We found the largest significant overlaps for hypomethylated DMPs with histone 3 lysine 27 tri-methylation (H3K27m3) and lysine 4 mono-methylation (H3K4m1) in MEFs [36] (Fig. 1e, f). These marks are indicative for repressed promoter regions and poised transcriptional enhancers, respectively. Smaller, significant overlaps were found for histone 3 lysine 4 tri-methylation (H3K4m3) in combination with H3K27m3 (bivalent chromatin), and for H3K4m1 in combination with histone 3 lysine 27 acetylation (H3K27ac) (active enhancer regions). No significant overlap was found with hypermethylated positions. These results suggest that many of the hypomethylated DMPs lie in regions involved in transcriptional regulation, which are however inactive in wt MEFs.

### Re-expression of ERβ complements hyper- but not hypomethylation

**Fig. 2:**
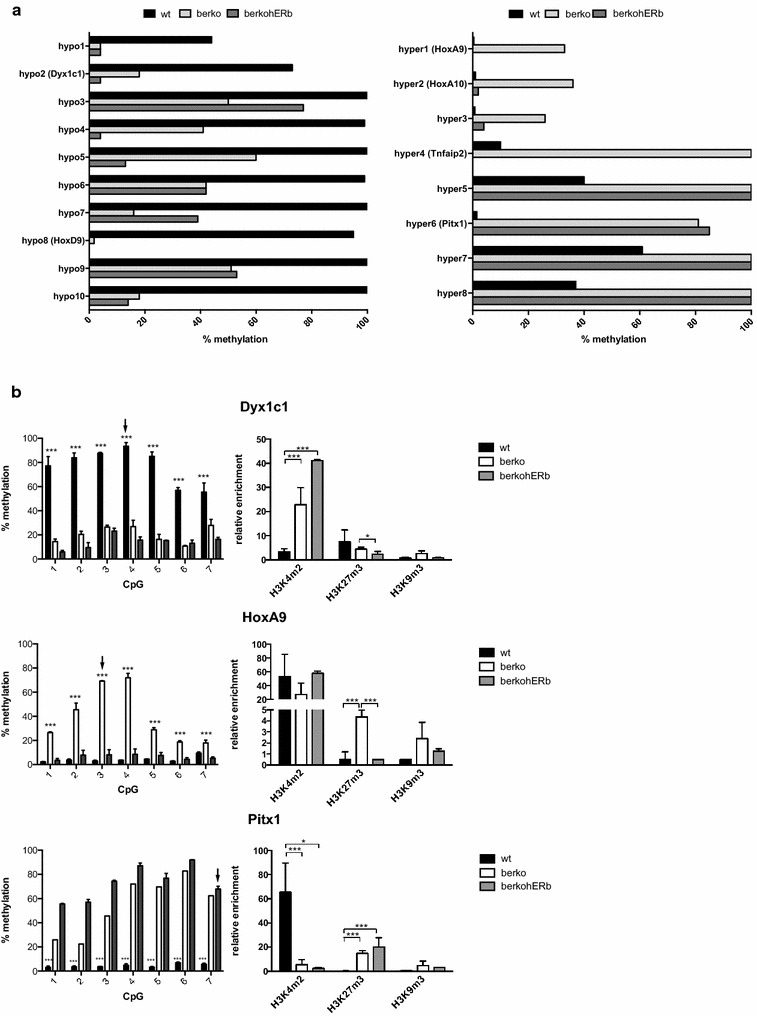
Hyper- but not hypomethylation in βerko MEFs is reversible by re-introduction of ERβ into βerko MEFs. **a** DNA methylation analysis of ten hypo- and eight hypermethylated positions. DNA methylation was assessed by methylation-specific enzymatic digest followed by qPCR. Positions with gene names in *brackets* were chosen for further analysis. **b** DNA methylation (*left panel*) and histone modifications (*right panel*) of differentially methylated genes in wt, βerko, and βerkohERβ MEFs. DNA methylation was assessed by pyrosequencing of bisulfite-treated DNA; *black arrows* mark DMPs identified by RRBS. *Triple* *Asterisk* indicates significant differences (*p* < 0.005) for wt vs. βerko and βerkohERβ (*Dyx1c1* and *Pitx1*) or βerko vs. wt and βerkohERβ (*HoxA9*). Histone modifications were analysed using ChIP followed by qPCR and normalised to *HPRT* (H3K4m2 and H3K27m3) or *GAPDH* promoter (H3K9m3) (means + SD; *n* ≥ 3)

We selected ten hypo- and ten hypermethylated DMPs with different genomic position and histone methylation patterns (Table 3). First, we analysed DNA methylation at DMPs by methylation-dependent restriction digest followed by real-time PCR in wt and βerko MEFs as well as in βerko MEFs complemented with an ectopically expressed ERβ cDNA (βerkohERβ) (Fig. 2a). We confirmed hypomethylation for all ten hypomethylated DMPs in Table 3 (Fig. 2a, left panel) and hypermethylation for eight of the ten hypermethylated DMPs (Fig. 2a, right panel). Re-introduction of ERβ restored wt methylation at a subset (4 of 8) of hypermethylated DMPs (Fig. 2a, right panel, DMP hyper1–4) but not at hypomethylated DMPs.

**Table 3:** Features of validated differentially methylated positions (DMPs)

DMP	Genomic location	CGI	Promoter MEFs	Promoter ESCs	Enhancer MEFs	Enhancer ESCs
Hypo1	Intergenic	No	H3K4m3/H3K27m3	H3K4m3	H3K4m1	
Hypo2 (Dyx1c1)	Promoter region	Yes	H3K4m3	H3K4m3		
Hypo3	Intragenic (intron)	No			H3K4m1	
Hypo4	Intragenic (intron)	No			H3K4m1	
Hypo5	Intragenic (intron)	No	H3K4m3		H3K4m1/H3K27ac	H3K4m1
Hypo6	Promoter region	No	H3K27m3	H3K27m3	H3K4m1	H3K4m1/H3K27ac
Hypo7	Intragenic (intron)	Yes	H3K4m3/H3K27m3	H3K27m3		H3K4m1
Hypo8 (HoxD9)	Promoter region	Yes	H3K4m3/H3K27m3	H3K4m3/H3K27m3	H3K4m1	
Hypo9	Promoter region	Yes	H3K4m3/H3K27m3	H3K4m3	H3K4m1/H3K27ac	
Hypo10	Promoter region	Yes	H3K4m3/H3K27m3	H3K4m3/H3K27m3	H3K4m1/H3K27ac	H3K4m1
Hyper1 (HoxA9)	Promoter region	No	H3K4m3	H3K4m3/H3K27m3		
Hyper2 (HoxA10)	Promoter region	No	H3K4m3/H3K27m3	H3K27m3		H3K4m1
Hyper3	Promoter region	No	H3K4m3/H3K27m3	H3K27m3		H3K4m1
Hyper4 (Tnfaip2)	Promoter region	Yes	H3K4m3	H3K4m3	H3K4m1	H3K4m1
Hyper5	Intragenic (intron)	No				
Hyper6 (Pitx1)	Promoter region	Yes	H3K4m3/H3K27m3	H3K4m3/H3K27m3		H3K4m1
Hyper7	Intragenic (intron)	No				
Hyper8	Intergenic	Yes	H3K4m3	H3K4m3	H3K4m1	

Genomic location, presence of a CpG island (CGI), and comparison with datasets for histone modifications enriched at promoter or enhancer regions in MEFs and embryonic stem cells (ESCs) using GenomeInspector (Genomatix). Hypo and hyper refer to hypo- and hypermethylation, respectively

Next, we analysed DNA methylation at regions surrounding DMPs (within 200–400 bp) and the effect of the ERβ agonist DPN on the methylation by pyrosequencing. Further, chromatin marks histone 3 lysine 4 dimethylation (H3K4m2) lysine 27 tri-methylation (H3K27m3) and lysine 9 tri-methylation (H3K9m3) around the DMPs was assessed by chromatin immunoprecipitation (ChIP). We found that the DNA methylation patterns at adjacent CpGs were similar to the ones at the identified DMPs, with one exception (shown in Fig. 2b and Additional file 1). DPN treatment, even for 4 days, had no effect on the DNA methylation pattern (Additional file 1). Histone modifications in the analysed regions matched the DNA methylation patterns: hypomethylated genes, exemplified by *Dyx1c1* [GenBank: NM_026314] in Fig. 2b, showed enrichment compared to a control region of both H3K4m2 and H3K27m3, reflecting a bivalent chromatin state, whereas hypermethylated genes, *HoxA9* [GenBank: NM_010456] and *Pitx1* [GenBank: NM_011097], displayed only enrichment for H3K4m2 in wt MEFs. This pattern was inverted in βerko MEFs, and complemented in βerkohERβ MEFs for *HoxA9* for which DNA methylation was complementable (Fig. 2b).

### ERβ regulates transcription of differentially methylated targets

**Fig. 3:**
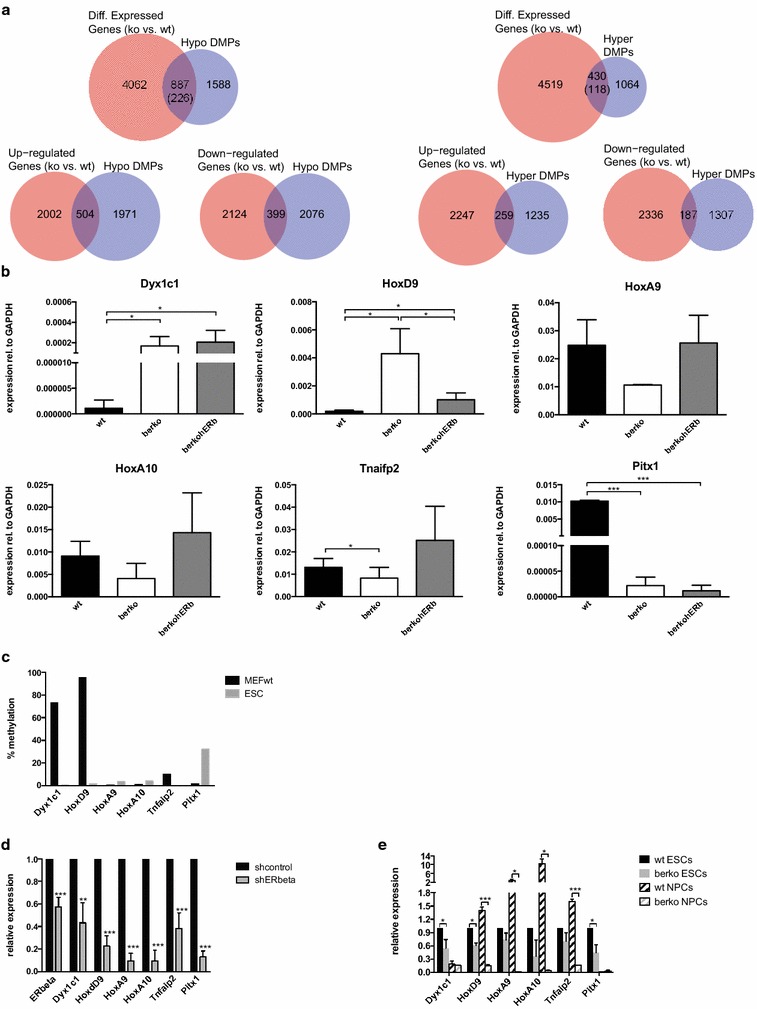
ERβ-dependent transcription of differentially methylated genes in MEFs and ESCs. **a** Venn diagram visualising overlaps between differentially methylated (identified by RRBS) and differentially expressed (identified by microarray expression analysis) genes in wt and βerko cells. **b** Gene expression analysis of hypomethylated (*Dyx1c1*, *HoxD9*), hypermethylated complementable (*HoxA9, HoxA10,* and *Tnfaip2*), and hypermethylated non-complementable genes in wt, βerko, and βerkohERβ MEFs. Gene expression was analysed by RT-qPCR (mean + SD; *n* ≥ 3). **c** DNA methylation of differentially methylated genes in wt MEFs and ESCs, assessed by methylation-specific enzymatic digest followed by qPCR. **d** ERβ-dependent expression of differentially methylated genes in ESCs. Gene expression was assessed by qRT-PCR 4 days after transfection with plasmid encoding for shRNA against ERβ or non-targeting control (means + SD; *n* ≥ 3). All the genes showed significantly decreased expression compared to shcontrol (** *p* < 0.01, *** *p* < 0.005). **e** ERβ-dependent expression of differentially methylated genes in wt and berko ESCs and NPCs derived thereof. Gene expression was assessed by qRT-PCR (means + SD; *n* ≥ 3; * *p* < 0.05, *** *p* < 0.005)

To investigate if differential methylation is associated with transcriptional changes, we compared gene expression in wt, βerko, and βerkohERβ MEFs using the Affymetrix^®^ Mouse Gene 1.1. ST platform. In total, we identified 4949 unique genes that showed a change in expression between wt and βerko MEFs (listed in Additional file 2). By re-introducing ERβ, 2051 genes showed differential gene expression compared to βerko MEFs (listed in Additional file 3). Two thousand five hundred and six genes were up-regulated, i.e. showed higher expression in βerko than in wt, and 2523 genes were down-regulated, i.e. showed lower expression in βerko than in wt. The genes nearest to DMPs (1494 unique genes for hypermethylated DMPs and 2475 for hypomethylated DMPs) were than compared to the differentially expressed genes. Twenty-nine percent (17 % up- and 12 % down-regulated) of the genes closest to hypermethylated DMPs and 36 % (20 % up- and 16 % down-regulated) of the ones closest to hypomethylated DMPs showed differential expression in βerko compared to wt MEFs (Fig. 3a). For both the hyper- and the hypomethylated genes, the overlap was bigger with up- than with down-regulated genes (17 vs. 12 % and 20 vs. 16 %, respectively). The expression of around 27 and 25 % of the genes overlapping with hyper- and hypomethylated genes, respectively, was rescued by re-introducing ERβ into the βerko MEFs (Fig. 3a).

For validation and further analysis of the relationship between methylation and transcription, we chose six genes that were identified as differentially expressed between wt and βerko MEFs and validated as differentially methylated in Fig. 2a: *Dyx1c1*, *HoxD9* [GenBank: NM_013555], *HoxA9, HoxA10* [GenBank: L08757], *Tnfaip2* [GenBank: NM_009396], and *Pitx1.* Transcription levels measured by qPCR were inversely correlated with the DNA methylation in MEFs; *Dyx1c1* and *HoxD9* showed higher expression in βerko and βerkohERβ cells, and *HoxA9, HoxA10, Tnfaip2,* and *Pitx1* higher expression in wt cells (Fig. 3b). As observed for DNA methylation, wt gene expression was restored by re-introduction of ERβ for *HoxA9, HoxA10,* and *Tnfaip2* (Fig. 3b). No effect of the ERβ ligand DPN was found on the expression of the tested genes (Additional file 1).

We also wanted to know if these genes are transcriptionally regulated by ERβ independently of their methylation status. Thus, we turned to mouse ESCs where DNA methylation is generally low. Indeed, DNA methylation was low at the investigated DMPs in ESCs (Fig. 3c). To test whether ERβ is involved in the transcriptional regulation of DMP-associated genes in ESCs, we assessed their expression in the absence and presence of ERβ in ESCs using small hairpin (sh)RNA mediated knocked-down of *ERβ*. As shown in Fig. 3d, knock-down of *ERβ* resulted in decreased expression of all of the tested genes. These results were in large parts confirmed in ESCs derived from βerko mice (Fig. 3e). Further, when these cells were differentiated towards neuronal precursor cells (NPCs), the effect of *ERβ* deficiency became larger, but only in those targets whose expression increased in NPCs (Fig. 3e). Genes that became silenced upon differentiation lost their ERβ dependency. Thus, all investigated targets identified as differentially methylated between wt and βerko MEFs are regulated by *ERβ*, but this is dependent on the cell type.

### ERβ binds to regions around DMPs

**Fig. 4:**
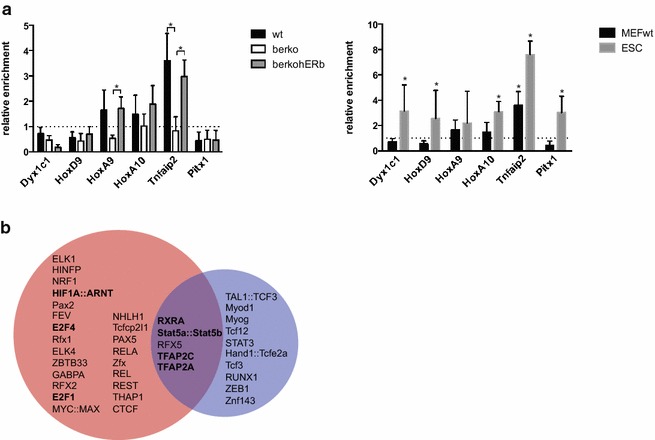
ERβ binds to regions around DMPs. **a** ERβ recruitment to differentially methylated genes in MEFs (*left panel*) and ESCs (*right panel*). HA-tagged ERβ was precipitated and differentially methylated regions were analysed by qRT-PCR. *Asterisk* indicates significant (*p* < 0.05) differences between wt/βerkohERβ and βerko MEFs or significant (*p* < 0.05) enrichment compared to binding at an unrelated, heterochromatic region on chromosome 2 [13] (means + SD; *n* ≥ 3). **b** Venn diagram visualising the enriched transcription factor binding motifs around hypo- or hypermethylated DMPs or both. Motifs that have been seen enriched in ERβ-ChIP-seq studies or that are bound by transcription factors known to interact with ERs are depicted in *bold*

To investigate direct involvement of ERβ in regulating these genes, we assessed the association of ERβ with regions around DMPs by ChIP in MEFs as well as in ESCs (Fig. 4a). ERβ was enriched in wt and βerkohERβ MEFs compared to βerko MEFs and an unrelated control region at these hypermethylated loci where re-introduction of ERβ was capable of reversing hypermethylation and increase expression of the associated gene (Fig. 4a, left panel). No enrichment was measured at hypomethylated loci in MEFs, but was evident in ESCs. This corroborates the findings that ERβ deficiency in ESCs leads to decreased transcription of these genes.

We next investigated if there are classical ER binding sites, EREs situated around the identified DMPs. Somewhat surprisingly, we could not identify any EREs in the regions we found ERβ-enrichment using ChIP. However, the hypermethylated loci where re-introduction of ERβ was capable of reversing hypermethylation had two motifs in common: Myog and activator protein 2 (AP2). The latter has been found enriched at ERβ binding sites in a breast cancer cell line using ChIP-seq [37]. The hypomethylated loci shared a binding motif for the regulatory factor for X-box 1 (Rfx1). No interaction between this factor and ERs has been described; however, interestingly, the family of Rfx transcription factors is involved in regulation and reading of DNA methylation marks [38–40].

Motif enrichment analysis of regions around all DMPs identified 636 potential EREs (genomic positions are listed in Additional file 4); however, no enrichment for ERE motifs was found compared to the reference genome. On the other hand, significant enrichment for binding sites of other transcription factors was identified (Fig. 4b, links to haystack motif enrichment analyses are provided in Additional files 5 and 6). Among these motifs, AP2, E2F, NRF1, and CTCF recognition sites had been shown to be enriched at ERβ binding sites [37]. Further, motifs were enriched for transcription factors known to interact with ERβ: Stat5 [41], RXRα [42], and ARNT [43]. Hyper- and hypomethylated loci showed enrichment for different motifs but shared binding sites for RXRα heterodimers, Stat5a::Stat5b heterodimers, RFX5, and AP2 (Fig. 4b).

Together, these results suggest that ERβ binds to the analysed differentially methylated regions not via classical EREs but via other binding sites or transcription factors.

### ERβ interacts with thymine DNA glycosylase

**Fig. 5:**
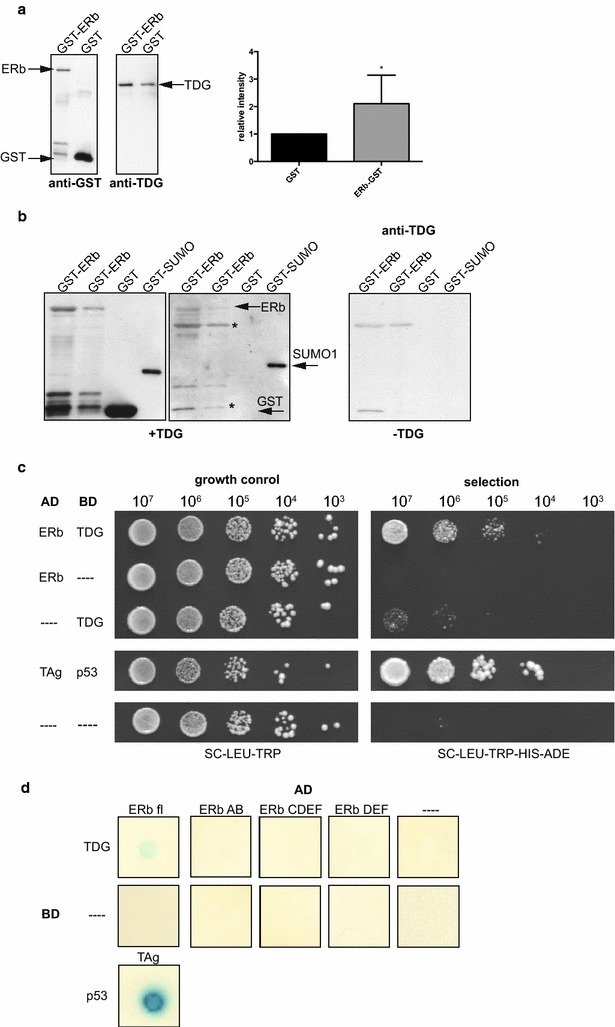
ERβ interacts directly with TDG. **a** Interaction of ERβ and TDG in GST-pulldown assays. GST-tagged ERβ or GST alone was immobilised on Glutathione Sepharose and incubated with recombinant TDG. The *left panel* shows representative western blots of the eluates using an anti-GST and an anti-TDG antibody. Quantification of four independent experiments is shown the right panel. *Asterisk* indicates significantly (*p* < 0.05) increased intensity of the band corresponding to TDG in the presence of ERβ. **b** Interaction of ERβ and TDG on far-western blots. GST-tagged ERβ (*lanes 1* + *2*), GST (*lane 3*), and GST-tagged SUMO-1 as a positive control (*lane 4*) were immobilised on a membrane and probed with recombinant TDG. Proteins were detected using antibody against GST (*left panel*) or TDG (*middle panel*). The *right panel* shows a membrane not probed with recombinant TDG. *Asterisk* marks unspecific bands. **c** Interaction between ERβ and TDG in yeast two-hybrid assays. ERβ fused to the AD and TDG fused to the BD of *GAL4* were expressed in the yeast strain AH109. Serial dilutions of cells were spotted on control (SC-LEU-TRP, *left panel*) and selective medium (SC-LEU-TRP-HIS-ADE, *right panel*) to monitor activity of the reporter genes *ADE2* and *HIS3*. As a positive control, murine p53 fused to *GAL4* BD was used in combination with SV40 large T-antigen fused to *GAL4* AD. The Gal4 BD and/or GAL4 AD alone served as negative controls (—). **d** Domain mapping for ERβ using yeast two-hybrid assays. Activity was tested in the yeast strain Y187 using *lacZ* as a reporter gene. Activity of the *lacZ*-encoded β-galactosidase leads to cleavage of X-gal and concomitant accumulation of a blue product (5,5′-dibromo-4,4′-dichloro-indigo). In addition to constructs as in B, individual ERβ domains (AB, CDEF, DEF) were fused to the *GAL4* AD and used for transformation of Y187 cells. 10^6^ cells were dropped onto SC plates lacking leucine and tryptophan. After 24 h of growth, cells were lysed and incubated with X-Gal for up to 17 h to monitor appearance of *blue colour*

Next, we investigated how ERβ can regulate DNA methylation at these specific sites. We hypothesised that ERβ may target factors regulating DNA methylation to these loci. ERα was previously shown to interact with TDG [44], an essential component of active DNA demethylation [13, 15, 16, 45]. We thus examined if ERβ can interact with TDG [GenBank: NM_172552, Swiss-Prot: P56581] as well. To this end, we conducted GST-pulldown assays using ERβ coupled to glutathion-S-transferase (GST) that was immobilised to Glutathione Sepharose and incubated with recombinant TDG. GST alone was used as a negative control. As shown in Fig. 5a, more TDG was recovered from GST-ERβ bound Sepharose compared to GST only. Quantification of four experiments showed that this increase in TDG recovery was statistically significant (Fig. 5a, right panel). However, there was still a considerable amount of unspecific binding of TDG to the matrix. Thus, we complemented these experiments with far-western blot analyses, immobilising the GST-tagged ERβ to nitrocellulose membrane and probing this membrane with recombinant TDG. Subsequent probing with a specific antibody against TDG highlighted a protein co-migrating with GST-ERβ but not with the GST-tag alone. GST-tagged SUMO-1, which has been shown to interact with TDG [46], was used as a positive control. As only unspecific bands were detected on a membrane that had not been probed with TDG (Fig. 5b, right panel), these results indicated a physical interaction of ERβ with TDG.

To corroborate these biochemical assays, we performed yeast two-hybrid assays in the *Saccharomyces cerevisiae* strain AH109. This strain harbours the two Gal4-inducible reporter genes *HIS*3 and *ADE*2 and protein interactions can be assessed by growth on selective medium lacking adenine and histidine. As expression of TDG fused to the *GAL4* activation domain (AD) induced some auto-activation of the reporter genes, as previously observed (data not shown), we addressed potential interactions by expressing ERβ fused to the *GAL4* AD and TDG fused to the *GAL4* DNA binding domain (BD). As shown in Fig. 5c, co-expression of ERβ and TDG enabled growth on selective medium. In contrast, little or no growth was detected when either factor was combined with the corresponding vector control, indicative of specific interactions between ERβ and TDG. This result was confirmed using another yeast strain (Y187) in which the *lacZ* gene, coding for the β-galactosidase, serves as the reporter gene to detect protein interactions (Fig. 5d). In addition to full-length ERβ, isolated domains of the receptor were tested to delineate the domain(s) responsible for interaction with TDG. We tested the AB, DEF, and CDEF domains of ERβ; however, no reporter activity above background could be detected for the interaction of TDG with any of these constructs (Fig. 5d). Together, these data provide strong evidence for a physical interaction between ERβ and TDG that requires the interaction of several domains of the receptor.

### TDG is a transcriptional co-activator of ERβ and is recruited to DMPs in an ERβ-dependent manner

**Fig. 6:**
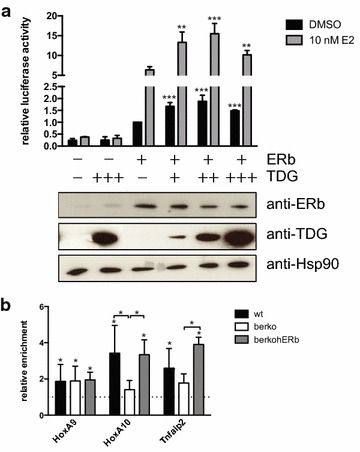
ERβ–TDG interaction affects gene regulation. **a** TDG increases ERβ transcriptional activity in reporter assays. Tdg^−/−^ MEFs were transfected with plasmids encoding for the reporter gene 3× ERE-luc, TK-renilla, ERβ, and TDG in different concentrations (50, 150, and 300 ng transfected plasmid), and treated with 10 nM E2. After 16 h, firefly luciferase activity was measured and normalised against renilla luciferase activity (means + SD; *n* ≥ 3). TDG co-expression increased luciferase activity significantly (** *p* < 0.01, *** *p* < 0.005). Expression of ERβ and TDG was confirmed by western blot analysis. **b** TDG is recruited to ERβ-regulated genes in MEFs. TDG recruitment to indicated genes in wt, βerko, and βerkohERβ MEFs was analysed by ChIP-qPCR. *Asterisk* indicates significant (*p* < 0.05) enrichment compared to binding at an unrelated, heterochromatic region on chromosome 2 [13] (means + SD; *n* ≥ 3)

To test if the interaction with TDG has an effect on ERβ function, we conducted reporter gene assays, measuring ERβ transcriptional activity. To this end, *Tdg*^−*/*−^ MEFs were co-transfected with plasmids encoding ERβ, a luciferase reporter gene driven by 3 EREs [47], and TDG. Four hours after transfection, cells were stimulated with E2 and harvested the next day for measurement of luciferase activity. Transfection of ERβ plasmid enhanced transcription of the reporter gene, and E2 treatment led to a further increase of luciferase activity (Fig. 6a). Co-transfection with TDG vector enhanced transcriptional activity of ERβ additionally. This increase was observed both in the absence (maximal 2-fold) as well as in the presence (maximal 2.5-fold) of ligand (Fig. 6a).

We then asked if TDG is recruited to the differentially methylated loci at which ERβ is enriched in wt MEFs. Using ChIP assays, we found TDG recruitment to these genes in wt and βerkohERβ MEFs (Fig. 6b). Notably, TDG enrichment at *HoxA10* and *Tnfaip2* appeared to be dependent on ERβ as it was not detectable in βerko MEFs, indicating that ERβ recruits TDG to these loci.

### TDG regulates genes associated with DMPs in ESCs

**Fig. 7:**
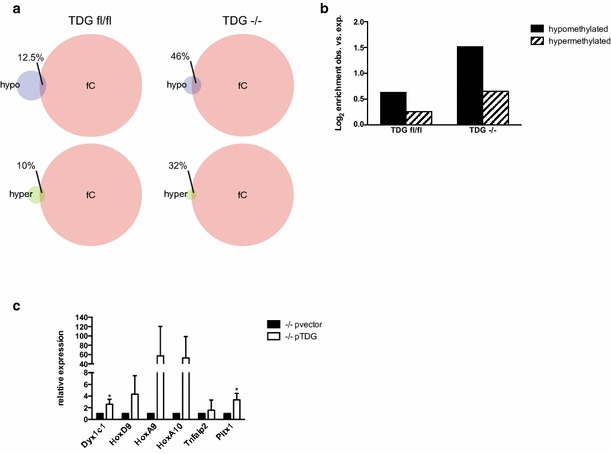
TDG regulates genes associated with DMPs in ESCs. **a** Venn diagram showing the overlap of hypo- and hypermethylated DMPs in βerko MEFs identified by RRBS with datasets for 5fC enrichment in deficient (*Tdg* ^−*/*−^) or proficient (*Tdg* ^*fl/fl*^) ESCs [16] using GenomeInspector (Genomatix). Hypo and hyper refer to the hypo- and hypermethylated regions identified in this study, respectively. **b** Enrichment (log2 ratios of observed over random) of 5fC in deficient (*Tdg* ^−*/*−^) or proficient (*Tdg* ^*fl/fl*^) ESCs at differently methylated positions. **c** TDG dependency of differentially methylated genes in ESCs. Gene expression in TDG-deficient (*Tdg* ^−/−^ pvector) and TDG-complemented (*Tdg* ^−/−^ pTDG) cells was assessed by qRT-PCR (means + SD; *n* = 3). *Asterisk* indicates significant (*p* < 0.05) increase in TDG-proficient vs. TDG-deficient cells

The proposed role for TDG in DNA demethylation is to process 5caC and 5fC [11]. Indeed, TDG-deficient ESCs show accumulation of 5fC and 5caC at gene regulatory elements [15, 16]. We therefore reasoned that if ERβ recruits TDG to certain genomic regions to regulate DNA methylation, the loss of ERβ would result in less TDG recruitment and, hence, accumulation of 5fC and 5caC at these loci. Comparison of genome-wide 5fC data [16] with our RRBS data revealed an overlap of hyper- and hypomethylated DMPs with 5fC in wt ESCs of 10 and 12.5 %, respectively. However, in TDG-deficient cells, this overlap increased to 32 and 46 % (Fig. 7a), which corresponds to a 1.6-fold and 2.9-fold of observed vs. expected enrichment, respectively (Fig. 7b). This indicates that nearly half of the hypomethylated DMPs identified in our screen overlapped with regions where TDG processes 5fC. Thus, we tested if TDG also transcriptionally regulates the differentially methylated targets in ESCs. Gene expression was analysed in TDG-deficient ESCs and cells complemented with TDG. A clear down-regulation was observed in ESCs lacking TDG (Fig. 7c), demonstrating that they are regulated by TDG.

## Discussion

Several studies suggest that NRs including the ERs are directly involved in regulating DNA methylation [25–30]. In this study, we systematically addressed the role of ERβ in regulating DNA methylation at specific loci. To this end, we carried out RRBS comparing ERβ-proficient and ERβ-deficient MEFs. Using this method, we limited the screen to CpG-rich regions and did not discriminate between 5mC and 5hmC modifications. In the analysis, we only considered DMPs that were fully methylated (80–100 %) in one cell type and unmethylated (0–20 %) in the other. Despite these constraints, we could identify more than 8000 DMPs, one-third of which was hyper- and two-thirds were hypomethylated. Around 30 % of the genes closest to hypo- or hypermethylated DMPs were differentially expressed between ERβ-proficient and ERβ-deficient cells, and expression of about one-third of them was rescued by re-expressing ERβ. Correlation between DNA methylation and expression changes was also demonstrated for selected genes associated with DMPs. Further, we found that they are bound and transcriptionally regulated by ERβ when they were actively transcribed. Further, we demonstrated that ERβ interacts physically and functionally with TDG, and that TDG associates with DMPs, in some cases ERβ-dependently, and is involved in the regulation of associated genes.

Based on our results, we propose that ERβ binds to regulatory regions of target genes and recruits TDG to these places. This interaction enhances gene expression on one hand and prevents DNA methylation on the other hand (illustrated in Additional file 7). The latter can be envisaged as the result of an interplay between TDG and the TET proteins that oxidise methylated CpGs to 5fC and 5caC, which in turn can be processed by TDG and the base excision repair to unmethylated C. Lack of ERβ results in less TDG recruitment and, hence, changes in gene transcription and DNA methylation, both hyper- and hypomethylation depending on the activity state of the respective gene. Hypermethylated DMPs were associated with genes actively transcribed in MEFs whose expression and methylation patterns could be restored upon re-introduction of ERβ into knock-out cells. This implicates an active demethylation mechanism involving TDG, as described previously for other genes in somatic cells [27, 28, 48, 49]. Hypomethylated genes, on the other hand, were inactivated in MEFs but transcribed and regulated by ERβ and TDG in ESCs, and hypomethylated DMPs overlapped remarkably with loci where 5fC accumulated in TDG-deficient ESCs [16]. As this mark is not recognised by the maintenance DNMT, 5fC accumulation leads to passive demethylation during cell division. Thus, at genes that become silenced during differentiation from ESCs to MEFs, lack of ERβ, and hence diminished TDG recruitment, could lead to erroneous passive demethylation, resulting in hypomethylation in the differentiated cells. This is corroborated by the findings that hypomethylated DMPs overlap with repressive chromatin marks in MEFs (Fig. 1e, f), that associated genes are involved in embryonic development (Table 2), and that genes that become repressed in NPCs are not regulated by ERβ anymore (Fig. 3e). At this point, we do not have an explanation for the occurrence of hypermethylated positions whose methylation pattern is not revertible by re-expression of ERβ other than clonal differences between wt and βerko cells.

Notably, as in our previous study [50], we could not find any effect of oestrogen on ERβ’s function at the loci investigated, neither on its transcriptional activity nor on its effects on DNA methylation and interaction with TDG. In contrast, TDG was shown to interact with ERα and enhance its transcriptional activity, in the presence of ligand [44]. The interaction between ERα and TDG is mediated by SRC-1, and overexpression of this co-factor in COS1 cells resulted in enhancement of ERα’s ligand-independent transcriptional activity by TDG [51]. Thus, availability of other factors involved in ER–TDG interaction in the different cell systems could explain the difference between the two receptor isoforms. On the other hand, ligand-independent function of ERβ has been shown previously in different contexts, e.g. when modulating ERα-induced gene expression in breast cancer cells [52]. Additionally, we have found that ERβ is tightly bound to the chromatin in extracts of different cell types even in the absence of ligand (our unpublished observation).

We could neither identify any classical EREs in the regions that ERβ bound to, nor were EREs found enriched around differentially methylated sites. This suggests that ERβ binds to these regions in complex with other factors. Trans-recruitment of big transcription factor complexes has been shown to be important for functional enhancer regions [53]. Indeed, we could find enriched motifs for TF that are known to interact with ERs, such as Stat5, RXR, and ARNT. Further, we found motifs that have been shown to be enriched in an ERβ-ChIP-seq study [37]. These included AP2 motifs that we found on all the investigated hypermethylated targets whose methylation and expression was restored by re-expressing ERβ but not in the non-complementable ones. The selected hypomethylated DMPs had Rfx1 sites in common, and motifs for Rfx factors were enriched on a genome-wide level. To our knowledge, no interaction between Rfx and ERβ has been described but, interestingly, these transcription factors, as well as CTCF that is enriched at hypomethylated DMPs, are implicated in regulation and reading of DNA methylation marks [38–40, 54]. Additionally, Our results imply that the function ERβ exerts at the identified targets differs from the classical ligand-induced ER signalling pathway. How ERβ binds to these loci and if and how the identified factors interact with ERβ has to be determined in future studies.

Although the factors that regulate changes in DNA methylation patterns during cell differentiation are identified, how they are recruited to and regulated at specific genomic loci is still unclear. R loop formation at CpG islands has been shown to exclude DNMT3a and DNMT3b, hence preventing methylation of these structures [55]. On the other hand, it has been suggested that locus-specific de novo methylation is induced by recruitment of DNMTs by non-coding RNAs [20–22] or by proximal sequence elements providing for specific transcription factor binding [17]. The involvement of transcription factors in regulating DNA methylation patterns has also been shown at distal regulatory regions with low methylation (LMRs) [18, 19]. The data presented here further support the notion that transcription factors can target DNA methylation and demethylation events, and provide a mechanism underlying this role. We suggest that interaction with factors regulating DNA methylation patterns is not limited to ERβ but could be general principle applying for many sequence-specific transcription factors. Indeed, several studies report an association between NR-induced transcription and DNA methylation changes [27, 28, 49].

## Conclusion

The data presented here suggest that ERβ can modulate DNA methylation patterns at specific genomic loci by interaction with TDG. This implies an important regulatory function of ERβ during cell differentiation and could be part of a mechanism underlying epigenetic alterations observed after exposure to compounds disrupting ER function in early development. Further, it supports a general concept in which transcription factors regulate the DNA methylation state at specific regions in the genome.

## Methods

### Plasmids and antibodies

Mission shRNA against *ERβ* was obtained from Sigma–Aldrich. Expression plasmid for HA-tagged mERβ (pSSH25-mERβ) was constructed by inserting PCR-amplified *mERβ* cDNA into pSHH25 [56] using *Xho*I and *Blg*II restriction sites. pSSH25-TDG for mammalian expression of HA-fused TDG [56], pPRS220 for yeast expression of Gal4-AD-fused TDG [46], 3× ERE-luc [47], and pSG5hERβ [57] used in luciferase assays have been published. pRL-TK for normalisation of luciferase activity was purchased from Promega. GST-ERβ was constructed by cloning cDNA encoding for human ERβ into pGEX-6P-3 (GE Healthcare) using *Bam*HI and *Xho*I restriction sites. pACT2-ERβ was obtained by cloning cDNA encoding for human *ERβ* into pACT2 (Clontech) using *Sma*I and *Xho*I restriction sites. Antibodies used were as follows: rat monoclonal anti-HA (3F10) from Roche Applied Science, rabbit monoclonal anti-ERβ (05-824), rabbit polyclonal anti-H3K4m2 (07-030), and rabbit polyclonal anti-H3K27m3 (07-449) from Millipore, rabbit polyclonal anti-H3K9m3 (060-050) from Diagenode, and mouse monoclonal anti-Hsp90 (F-8) from Santa Cruz Biotechnology, Inc.

### Cell culture and transfections

MEFs from wt and βerko mice [25] and βerko MEFs complemented with ERβ (βerkohERβ [25]) as well as *Tdg*^*−/*−^ MEFs [56] were kept in high-glucose DMEM supplemented with 10 % FCS, 1 mM l-glutamine, 1 mM sodium pyruvate, and 1× non-essential amino acids and 5 μg/ml blasticidin (βerkohERβ). For stimulation with ER agonists, cells were put for at least 2 days into DMEM with 5 % dextran-coated charcoal-treated serum and treated with 10 nM E2 or DPN.

ESCs [58] were thawed on feeder cells in ESC medium (high-glucose DMEM supplemented with 15 % heat-inactivated FCS, 1 mM sodium pyruvate, 1× non-essential amino acids, and 0.1 mM β-mercaptoethanol) containing 1 U/μl LIF (Millipore). Upon feeder removal, cells were maintained in 2i medium (serum-free N2B27 [59] supplemented with 2i inhibitors [60], CHIR99021 (3 μM), and PD0325901 (1 μM), obtained from the division of signal transduction therapy, University of Dundee) containing 1 U/μl LIF. TDG knock-out ESCs complemented with TDG (*Tdg*^−*/*−^ pTDG) or empty vector (*Tdg*^−*/*−^ pvector) were maintained in 2i medium containing 1 μg/ml puromycin.

NPCs were generated from ERβ^+/+^and ERβ^−/−^ ESCs, kindly contributed by Prof. Jan-Åke Gustafsson, via Embryoid Bodies (EBs). To generate EBs, feeder-independent mESCs were grown in serum-free 2i media for 2–3 passages and further split at a density of 5.5 × 10^5^ cells/ml in EB medium (15 % knoc-kout Serum Replacement, 2 mM l-Glutamine (200 mM), 10 mM HEPES (1 M), 0.1 mM non-essential amino acids MEM (10 mM), 0.1 mM β-mercaptoethanol (50 mM), 10 µg/ml Gentamicin (10 mg/ml) in knoc-kout DMEM [high glucose with sodium pyruvate) (Invitrogen)] in a 100-mm non-adherent dish (non-tissue culture treated). The medium was replaced daily for 3 days with taking care of not to disturb cell aggregates. EBs were grown for 3 days in suspension and collected in a 50-mL conical tube containing 10 mL fresh EB medium, supplemented with 15 % ES-qualified FBS. EBs were transferred to gelatin-coated tissue culture plates at a ratio of 1:10 by gentle pipetting and cultured for 24 h at 37 °C and 5 % CO_2_. Medium was replaced by ITS–Fibronectin medium (495 ml knock-out DMEM/F12 without HEPES, 775 mg glucose, 36.5 mg l-glutamine, 1.2 g NaHCO_3_ (Invitrogen), and 5 ml ITS media supplement 100×, R and D systems). Recombinant bovine fibronectin (R and D Systems) was added to this medium at 5 µg/ml concentration. Cells were cultured for 6–8 days at 37 °C and 5 % CO_2_. ITS–Fibronectin medium was changed every other day. During this period, a monolayer grew from the attached EBs. ITS/Fibronectin medium was removed from the cell culture and the attached cells were washed twice with 10 mL of sterile DPBS. Cells were dissociated with Accutase solution and collected in a 15-mL tube containing ITS–Fibronectin medium by gentle pipetting. Cell clumps were removed (remnants of EB) by allowing the tube to stand just long enough to allow the cell clumps to settle to the bottom (about 5 min). Suspended cells were transferred to a new 15-mL tube by gentle pipetting and centrifuged for 5 min at 1500 rpm to pellet the cells. Cell pellets were resuspended in MN3FL medium (495 ml knock-out DMEM/F12 without HEPES, 775 mg glucose, 36.5 mg l-glutamine, 845 mg NaHCO_3_ (Invitrogen), 5 ml N2-Max media supplement 100×, 10 ng/ml FGF basic (R and D systems), and 1 µg/ml Laminin, Novus Biologicals). Cells were plated on poly-l-ornithine/laminin-coated dishes at a seeding density of 60,000 cells/cm^2^. Cells were fed MN3FL medium every day for 4–6 days until cell confluency reached close to 100 %. MN3FL medium was removed from the cell culture and attached cells were washed with sterile DPBS and dissociated with Accutase solution and passaged on poly-l-ornithine/laminin-coated dishes. Selected NPCs were grown further up to 6–10 passages.

For transfection with HA-ERβ, MEFs or ESCs were seeded onto 15-cm plates and transfected the following day using JetPRIME reagent (Polyplus transfection) according to the manufacturer’s protocol. One day after transfection, cells were harvested for ChIP.

For transfection with shRNA constructs, ESCs were seeded into 6-well dishes and transfected the following day using JetPRIME reagent according to the manufacturer’s protocol. Twenty-four hours after transfection, 1 μg/ml puromycin was added to the medium. Transfected cells were selected for 4 days changing medium daily, and harvested for RNA extraction.

Transfection for luciferase assays was performed using JetPEI (Polyplus transfection) in 24-well plates with 50 ng pRL-TK, 50 ng pSG5-hERβ, 100 ng 3× ERE-luc, and varying concentration 50, 150, and 300 ng) of pSHH25-TDG per well, according to the manufacturer’s protocol.

### RRBS

Library preparation for RRBS was carried out as described [61]. In brief, genomic DNA derived from wt and βerko MEFs was isolated using the QIAamp DNA mini kit (Qiagen). Three batches of genomic DNA of each cell type were pooled, and 1 μg was digested with 20 U MspI overnight. The reaction was stopped by addition of 1 μl 0.5 M EDTA and purified using MinElute gel extraction kit (Qiagen). Subsequently, DNA fragments were end-repaired and A-tailed by incubation with 5 U Klenow fragment (New England Biolabs Inc.) and 0.5 mM dATP and 0.05 mM dGTP and dCTP at 30 °C for 20 min followed by 20 min at 37 °C. After purification using the MinElute gel extraction kit, methylated Illumina standard adapters were ligated to the fragments overnight at 16 °C using 400 U T4 ligase (New England Biolabs Inc.). The fragments were then separated on a 3 % Nusieve 3:1 agarose 0.5× TBE gel and 160–340 bp fragments were excised and purified using the MinElute gel extraction kit. The purified DNA was subjected to two rounds of bisulfite conversion using the EZ DNA methylation kit (Zymo Research). Subsequently, the final library was prepared by 19 cycles of PCR amplification and purification using the MinElute gel extraction kit. Libraries were sequenced on the Illumina Genome Analyser IIx following the manufacturer’s protocol at the D-BSSE, ETH Basel. These data have been deposited in NCBI's Gene Expression Omnibus [64] and are accessible through GEO Series accession number GSE72230 (https://www.ncbi.nlm.nih.gov/geo/query/acc.cgi?acc=GSE72230).

### RRBS methylation analyses

Mapping of obtained sequences was performed using the Genomatix mining station (http://www.genomatix.de/solutions/genomatix-mining-station.html). Annotation and correlation analyses were carried out using the Genomatix Regionminer. Statistical analyses were carried out using R [62] or GraphPad Prism. Statistical significance was assessed using the paired *t* test or Fisher exact test. The level of significance was selected as *p* < 0.05.

### Bisulfite treatment and pyrosequencing

Genomic DNA (200–500 ng) was bisulfite treated and purified using the EZ DNA Methylation kit (Zymo Research). One micro litre of the converted DNA was used for nested PCR amplification (for primer sequence see Additional file 8), and the PCR product was sequenced by pyrosequencing in a Pyromark Q24 (Qiagen).

### Methylation-sensitive restriction enzyme digest

Five micro gram of genomic DNA was digested with 50 U HpaII or 100 U MspI overnight. Subsequently, enzymes were removed by digest with proteinase K for 30 min at 40 °C and digested fragments were analysed by real-time PCR (primers listed in Additional file 8) using Rotor-Gene SYBR Green PCR Kit on a Rotor-Gene RG-3000 (Qiagen).

### RNA isolation, cDNA production, and real-time PCR

RNA was isolated using Tri (Sigma) according to the manufacturer’s recommendations. One microgram of total RNA was treated with DNAseI (New England Biolabs Inc.) and reverse transcribed using random hexamer primers (Fermentas). One microlitre of the resulting cDNA was used for real-time PCR using Rotor-Gene SYBR Green PCR kit on a Rotor-Gene RG-3000 (Qiagen). Gene transcripts were normalised to the *Gapdh* RNA content (primers listed in Additional file 8). All results are based on the ΔΔCT method and represent the mean of at least three independent experiments.

### Gene expression microarray analysis

Gene expression microarray data were generated using the Affymetrix^®^ Mouse Gene 1.1. ST platform. The data were processed using the Affy package with software R. Global background correction was performed through the robust multi-array average (RMA), and the statistical significance was calculated by the empirical Bayes model. The expression probes with adjusted *p* value less than 0.01 and with at least 0.5-fold change between two conditions are defined as differentially expressed probes.

### Motif enrichment analysis

Enriched transcription factor motifs as well as classical ERE motif finding were identified using Haystack software (PMID: 24395799) according to the manuals. Briefly, genomic regions containing 200 bp around the DMPs were selected for motif analysis and genomic regions containing 200 bp around the CpG sites that can be covered by RRBS experiments were chosen as the reference genome (background). The classical ERE motif was defined as the consensus sequence of GGTCAnnTGACC (PMID: 12824376).

### Chromatin immunoprecipitation

ChIP assays were performed as described [43] with minor modifications. Cells were grown to confluency on 15-cm dishes. Chromatin was cross-linked for 10 min with 1 % formaldehyde (Pierce Biotechnologies Inc.) and the reaction was stopped by addition of 125 mM glycine for 10 min. After washing twice with cold PBS, cells were harvested in PBS by centrifugation at 4 °C at 600*g*. Nuclei were isolated by sequential 5-min incubation on ice with 500 μl cold nucleus/chromatin preparation (NCP) buffer I (10 mM HEPES pH 6.5, 10 mM EDTA, 0.5 mM EGTA, 0.25 % Triton X-100) and twice cold NCP buffer II (10 mM HEPES pH 6.5, 1 mM EDTA, 0.5 mM EGTA, 200 mM NaCl). Pelleted nuclei were lysed in 200–400 μl lysis buffer [50 mM Tris–HCl pH 8.0, 1 mM EDTA, 0.5 % Triton X-100, 1 % SDS, 1 mM PMSF, 1× Complete (Roche)] for 10  min followed by sonication for 15 cycles (30 s on, 30 s off, power high) using a Bioruptor sonicator (Diagenode). After centrifugation at 4  °C and 14,000*g* for 10 min, chromatin concentration was estimated by absorbance at 260 nm. Hundred microgram (for HA, TDG and H3K9m3 ChIPs) or 50 µg (for H3K4m2 and H3K27m3 ChIPs) of chromatin were diluted ten times in IP buffer I (50 mM Tris–HCl pH 8.0, 1 mM EDTA, 150 mM NaCl, 0.1 % Triton X-100, 1 mM PMSF, 1× Complete) for HA and TDG ChIPs or IP buffer II (20 mM Tris–HCl pH 8.0, 2 mM EDTA, 150 mM NaCl, 1 % Triton X-100, 1 mM PMSF, 1× complete) for histone ChIPs. Diluted chromatin was pre-cleared at 4  °C for 1 h with 40 µl of a 50 % slurry of magnetic Protein G beads (Invitrogen) preblocked with 1 mg/ml BSA and 1 mg/ml tRNA. Pre-cleared chromatin was incubated with 2–5 µg of the respective antibody (listed in Additional file 9) overnight at 4  °C and immuno-complexes were precipitated with 40 µl of a 50 % slurry of blocked Protein G beads at 4  °C for 2 h. Subsequently, beads were serially washed with 500 µl wash buffer I (20 mM Tris–HCl pH 8.0, 2 mM EDTA, 150 mM NaCl, 0.1 % SDS, 1 % Triton X-100), 500 µl wash buffer II (20 mM Tris–HCl pH 8.0, 2 mM EDTA, 500 mM NaCl, 0.1 % SDS, 1 % Triton X-100), and 500 µl wash buffer III (10 mM Tris–HCl pH 8.0, 1 mM EDTA, 250 mM LiCl, 1 % sodium deoxycholate, 1 % NP-40). For TDG ChIPs, beads were washed once with 500 µl wash buffer I and twice with 500 µl wash buffer II. After two additional washes with 500 µl TE buffer (10 mM Tris–HCl pH 8.0, 1 mM EDTA), complexes were eluted by incubating twice with 250 µl extraction buffer (1 % SDS, 0.1 M NaHCO_3_) at 65  °C for 15 min. Crosslink was reversed by incubation at 65  °C for 4 h in the presence of 200 mM NaCl. Subsequently, proteins were removed by incubation with proteinase K (50 µg/ml) in the presence of 10 mM EDTA at 45  °C for 1 h, and DNA was purified by phenol/chloroform extraction and ethanol precipitation. The isolated DNA fragments were analysed by qPCR (primers listed in additional file 8) using Rotor-Gene SYBR Green PCR kit on a Rotor-Gene RG-3000 (Qiagen).

### GST-pulldown assays

GST-ERβ and GST were expressed in *E.coli* BL21 at 15 °C overnight upon induction with 0.2 mM IPTG. Cells were harvested and lysed in 2 ml GST buffer [10 mM Tris–HCl, pH 8; 50 mM NaCl; 5 % Glycerol; 1 mM DTT; 0.1 mM PMSF; 1× complete (Roche)] by sonication in a Bioruptor^®^ UDC-200 (Diagenode). After sonication, 1 % Triton X-100 was added to the lysates, and samples were gently mixed for 30 min at 4 °C and centrifuged at 12,000×*g* for 10 min at 4 °C. Lysates were centrifuged at 12,000×*g* for 10 min at 4 °C and supernatants transferred to fresh tubes and protein concentration was assessed using Bradford assay. Glutathione Sepharose high-performance beads (GE Healthcare) were prepared to 50 % slurry as described in the manufacturer’s protocol. The lysates were diluted in 500 µl GST buffer to a final total protein concentration of 0.8 µg/µl, 10 µl glutathione Sepharose 50 % slurry was added, and the mixture was incubated at 4 °C for 2 h with agitation. Three hundred and fifty nanogram recombinant hTDG was added, followed by incubation at 4 °C for 2 h with agitation. The beads were washed three times with GST washing buffer (10 mM Tris–HCl, pH 8; 80 mM NaCl; 5 % Glycerol) and once with GST wash buffer containing 150–300 mM NaCl. Proteins were eluted by boiling in Laemmli buffer at 95 °C for 5 min and analysed using SDS-PAGE followed by western blot. Band intensity was quantified using ImageJ.

### Western blot and far-western blot analyses

The insoluble fraction of luciferase assay-lysates containing chromatin-bound proteins was used for protein expression analysis by immunoblotting using anti-TDG, anti-ERβ, and anti-Hsp90 antibodies.

For far-western blot analysis, GST-tagged proteins were expressed as described above. GST-ERβ was purified by affinity chromatography using glutathione Sepharose (GE Healthcare) according to the manufacturer’s protocol. Proteins were eluted by boiling in Laemmli buffer at 95 °C for 5 min. Proteins were fractionated by SDS-PAGE and transferred to a nitrocellulose membrane. The proteins on the membrane were denatured by incubation with 6 M guanidine-HCl (GuHCl) in AC buffer (20 mM Tris–HCl, pH 7.6; 100 mM NaCl; 10 % Glycerol; 0.1 % Tween-20; 2 % skim milk powder; 1 mM DTT; 0.5 mM EDTA) for 30 min at RT, and renatured by washing steps with 3 M GuHCl for 30 min at RT, 1 M GuHCl for 30 min at RT, 0.1 M GuHCl for 30 min at 4 °C, and AC buffer only for 1 h at 4 °C. Upon blocking with 5 % skim milk powder in TBST for 1 h at RT, the membrane was incubated at 4 °C overnight with gentle shaking in protein-binding buffer (20 mM Tris–HCl, pH 8; 50 mM NaCl; 10 % Glycerol; 0.1 % Tween-20; 2 % skim milk powder; 1 mM DTT) containing 7–9 µg recombinant TDG [63]. The membranes were washed thoroughly 3 times with TBS containing 0.2 % NP-40. Subsequently, bound proteins were detected using antibodies against TDG and GST.

### Yeast two-hybrid analysis of ERβ–TDG interaction

The Matchmaker™ yeast two-hybrid system (Clontech) was used. Bait and trait proteins were cloned into plasmids encoding the binding and AD of the Gal4 protein, respectively. The *S. cerevisiae* strains AH109 (*MATa, trp1*–*901, leu2*–*3, 112, ura3*–*52, his3*–*200, gal4*Δ*, gal80*Δ, *LYS2::GAL1*_UAS_–*GAL1*_TATA_–*HIS3, MEL1, GAL2*_UAS_–*GAL2*_TATA_–*ADE2, URA3::MEL1*_UAS_–*MEL1*_TATA_–*lacZ)* and Y187 (*MATα, ura3*–*52, his3*–*200, ade2*–*101, trp1*–*901, leu2*–*3, 112, gal4*Δ*, met*^−^*, gal80*Δ*, URA3::GAL1*_UAS_–*GAL1*_TATA_–*lacZ*) were co-transformed with 50–500 ng of bait and trait plasmids according to the Clontech manual. For AH109, interactions were assessed by spotting serial dilutions of cells on selective medium (SC-LEU-TRP-ADE-HIS) and incubating them for 2–4 days at 30 °C. β-Galactosidase activity was assayed using the Y187 strain (Clontech manual). Briefly, 10^6^ cells were dropped on SC medium selecting for the plasmids (SC-LEU-TRP) and grown for 24 h at 30 °C. Cells were transferred to filter paper (Filtrak, 80 g/m^2^) before snap-freezing in liquid nitrogen and subsequent thawing for cell lysis. The filter with the lysed cells was soaked with 2 ml of Z buffer (100 mM Na phosphate buffer pH 7.0, 10 mM KCl, 1 mM MgSO_4_, 33 µM β-mercaptoethanol, 817 µM X-Gal) and incubated at 30 °C for up to 17 h.

### Luciferase assays

Four hours after transfection, 10 nM E2 was added to the medium. The next day, luciferase reporter assays were performed using the Dual-Luciferase^®^ Reporter Assay System (Promega) according to the manufacturer’s protocol: cells were lysed in passive lysis buffer (Promega) and firefly and renilla luciferase activity measured in 96-well plates using a luminometer Centro LB 960 (Berthold technologies).

## Additional files

10.1186/s13072-016-0055-7 Additional DNA methylation and gene expression analyses. A: DNA methylation of regions surrounding DMPs. DNA methylation of four additional differentially methylated genes in wt, βerko, and βerkohERβ MEFs, assessed by pyrosequencing of bisulfite-treated DNA. Black arrows mark DMPs identified by RRBS. B: Effect of the ERβ-agonist DPN on DNA methylation of identified target genes. DNA methylation of differentially methylated genes in wt, βerko, and βerkohERβ MEFs upon 0, 6, 24, and 96 h treatment with 10 nM DPN, assessed by pyrosequencing of bisulfite-treated DNA. C: Effect of the ERβ-agonist DPN on expression of identified target genes. Gene expression analysis in wt, βerko, and βerkohERβ MEFs upon a 0 and 6 h treatment with 10 nM DPN, analysed by RT-qPCR.
10.1186/s13072-016-0055-7 Genes differently expressed in wt and βerko MEFs. List of genes that show changed expression in βerko compared to wt MEFs assessed by microarray gene expression analyses using the Affymetrix^®^ Mouse Gene 1.1. ST platform. The list includes fold change, *p* value, chromosomal location, gene name and annotation.
10.1186/s13072-016-0055-7 Genes differently expressed in βerko and βerkohERβ MEFs. List of genes that show changed expression in βerkohERβ compared to βerko MEFs assessed by microarray gene expression analyses using the Affymetrix^®^ Mouse Gene 1.1. ST platform. The list includes fold change, *p* value, chromosomal location, gene name and annotation.
10.1186/s13072-016-0055-7 Genomic location of the identified EREs.
10.1186/s13072-016-0055-7 Links to enriched transcription factor motifs identified using Haystack software.
10.1186/s13072-016-0055-7 Links to enriched transcription factor motifs identified using Haystack software.
10.1186/s13072-016-0055-7 Model of ERβ-TDG interaction and its effects. ERβ binds to regulatory regions of target genes and recruits TDG to these places. This interaction enhances gene expression on one hand and affects DNA methylation on the other hand. The effects on the DNA methylation pattern depends on the activity of the respective gene in a given cell type.
10.1186/s13072-016-0055-7 Primers used for real-time PCR and Pyrosequencing. MSD: methylation sensitive restriction enzyme digest, pyro: pyrosequencing.
10.1186/s13072-016-0055-7 Antibodies used for western blotting and ChIP.

## Abbreviations

5caC: 5-carboxylcytosine
5fC: 5-formylcytosine
5hmC: 5-hydroxymethylcytosine
5mC: 5-methylcytosine
AD: activation domain
AP2: activator protein 2
ARNT: aryl hydrocarbon receptor nuclear translocator
BD: binding domain
BSA: bovine serum albumin
CGI: CpG island
ChIP: chromatin immunoprecipitation
DMEM: Dulbecco’s modified eagle medium
DMPs: differentially methylated positions
DNMT: DNA methyl-transferase
DPN: diarylpropionitrile
E2: 17-β-estradiol
EDTA: ethylene diamine tetraacetic acid
EGTA: ethylene glycol tetraacetic acid
ERα: oestrogen receptor α
ERβ: oestrogen receptor β
ERE: oestrogen response element
ESCs: embryonic stem cells
FCS: foetal calf serum
Glut4: glucose transporter 4
GO: gene ontology
GST: glutathion-S-transferase
H3K4m3: lysine 4 tri-methylation at histone H3
H3K27m3: lysine 27 tri-methylation at histone H3
H3K9m3: lysine 9 tri-methylation at histone H3
HA: hemagglutinin
IPTG: isopropyl-β-d-thiogalactopyranoside
LIF: leukaemia inhibitory factor
LMR: low methylated regions
MEFs: mouse embryonic fibroblasts
Myog: myogenin
NPC: neuronal precursor cell
NR: nuclear receptor
NRF1: nuclear respiratory factor-1
PMSF: phenylmethylsulfonyl fluoride
Rfx: regulatory factor for X-box
RRBS: reduced representation bisulfite sequencing
SDS: sodium dodecyl sulphate
shRNA: small hairpin RNA
RXR: retinoid X receptor
Stat: signal transducers and activators of transcription
SUMO: small ubiquitin-related modifier
TDG: thymine DNA glycosylase
TET: ten-eleven translocation
wt: wildtype
