# Supplementary figures and images for: Oestrogen receptor β regulates epigenetic patterns at specific genomic loci through interaction with thymine DNA glycosylase
Source: Epigenetics Chromatin. 2016 Feb 16;9:7. doi: 10.1186/s13072-016-0055-7 (PMC4756533; doi:10.1186/s13072-016-0055-7)

A

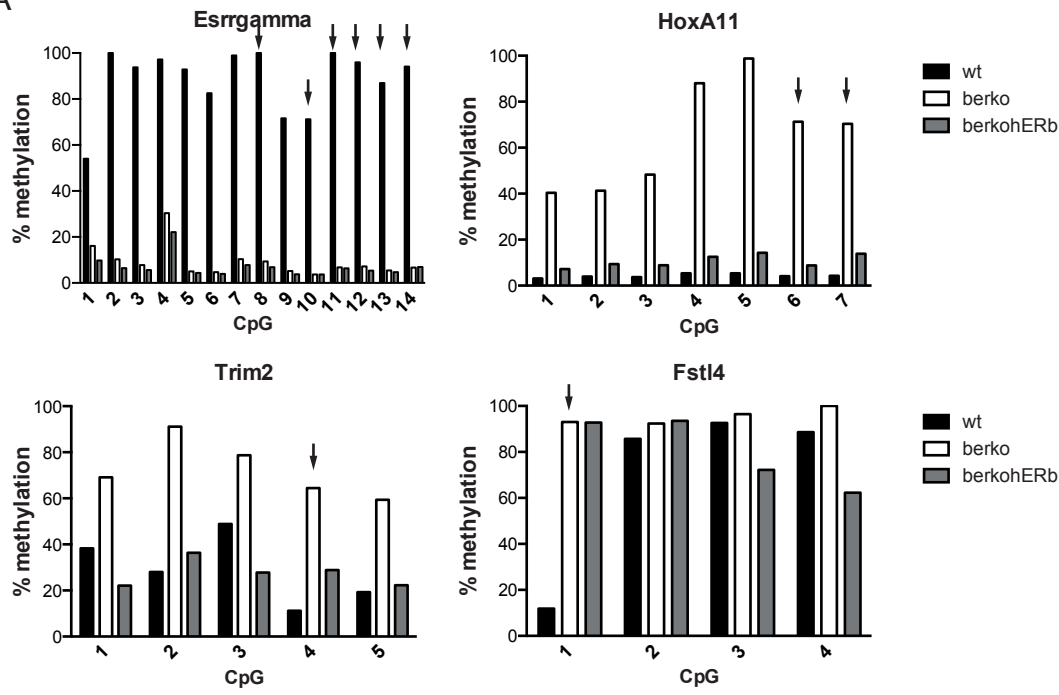

B

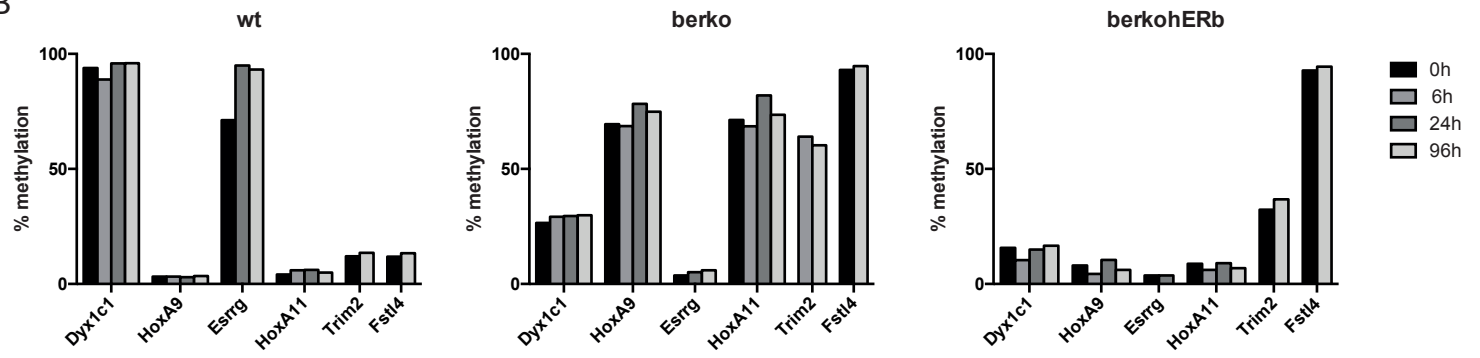

C

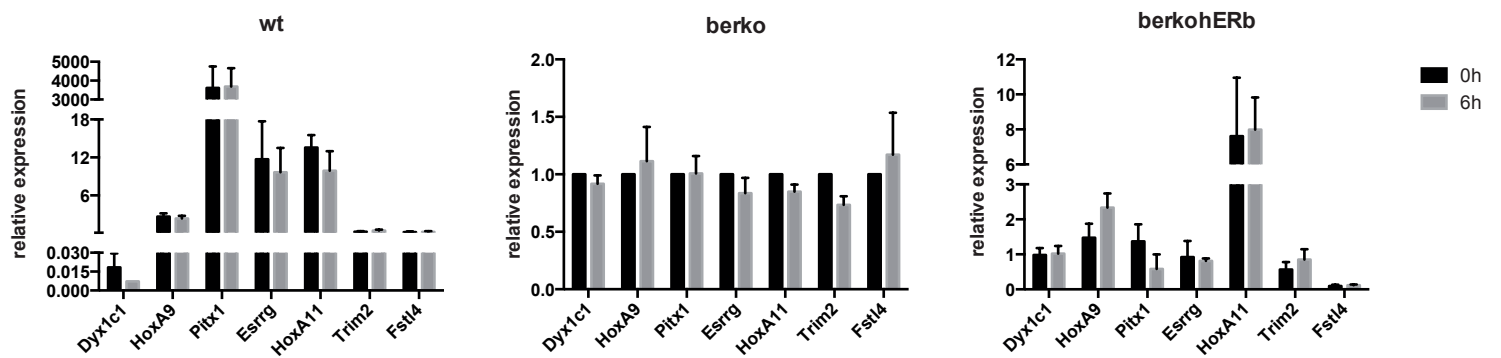

Supplement: Supplementary file 1 — 10.1186/s13072-016-0055-7 Additional DNA methylation and gene expression analyses. A: DNA methylation of regions surrounding DMPs. DNA methylation of four additional differentially methylated genes in wt, βerko, and βerkohERβ MEFs, assessed by pyrosequencing of bisulfite-treated DNA. Black arrows mark DMPs identified by RRBS. B: Effect of the ERβ-agonist DPN on DNA methylation of identified target genes. DNA methylation of differentially methylated genes in wt, βerko, and βerkohERβ MEFs upon 0, 6, 24, and 96 h treatment with 10 nM DPN, assessed by pyrosequencing of bisulfite-treated DNA. C: Effect of the ERβ-agonist DPN on expression of identified target genes. Gene expression analysis in wt, βerko, and βerkohERβ MEFs upon a 0 and 6 h treatment with 10 nM DPN, analysed by RT-qPCR. [file 13072_2016_55_MOESM1_ESM.pdf]

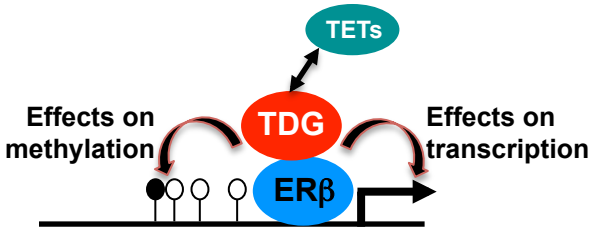

Supplement: Supplementary file 7 — 10.1186/s13072-016-0055-7 Model of ERβ-TDG interaction and its effects. ERβ binds to regulatory regions of target genes and recruits TDG to these places. This interaction enhances gene expression on one hand and affects DNA methylation on the other hand. The effects on the DNA methylation pattern depends on the activity of the respective gene in a given cell type. [file 13072_2016_55_MOESM7_ESM.pdf]
