# Supplementary material for: Oestrogen receptor β regulates epigenetic patterns at specific genomic loci through interaction with thymine DNA glycosylase
Source: Epigenetics Chromatin. 2016 Feb 16;9:7. doi: 10.1186/s13072-016-0055-7 (PMC4756533; doi:10.1186/s13072-016-0055-7)
Supplement: Supplementary file 5 — 10.1186/s13072-016-0055-7 Links to enriched transcription factor motifs identified using Haystack software. [file 13072_2016_55_MOESM5_ESM.html]

Haystack - Motif enrichment Analysis


Haystack Motif Enrichment Analysis Tool

Target Coordinates: ./hypo.bed (6016)
  
Background Coordinates: random\_background (12032)
  
Motifs database file:/opt/apps/Haystack/motif\_databases/JASPAR\_CORE\_2014\_vertebrates.meme
  
Command used: */opt/apps/Haystack/bin/haystack\_motifs ./hypo.bed mm9*

| Motif ID | Motif Name | Presence in Target | Presence in BG | Ratio | p-value | q-value | Central Enrichment | Motif Profile | Logo | Regions with Motif | Nearby Genes |
| --- | --- | --- | --- | --- | --- | --- | --- | --- | --- | --- | --- |
| MA0028.1 | ELK1 | 13.30% | 6.50% | 1.91 | 1.15e-49 | 2.87e-48 | 2.09 |  |  | list of regions | genes list |
| MA0131.1 | HINFP | 3.97% | 1.65% | 1.87 | 2.69e-20 | 3.36e-19 | 1.69 |  |  | list of regions | genes list |
| MA0506.1 | NRF1 | 37.70% | 25.37% | 1.47 | 1.83e-64 | 9.17e-63 | 1.55 |  |  | list of regions | genes list |
| MA0259.1 | HIF1A::ARNT | 9.13% | 5.93% | 1.46 | 6.29e-15 | 4.49e-14 | 1.29 |  |  | list of regions | genes list |
| MA0074.1 | RXRA::VDR | 5.12% | 3.46% | 1.37 | 1.55e-07 | 4.83e-07 | 1.14 |  |  | list of regions | genes list |
| MA0067.1 | Pax2 | 3.72% | 2.57% | 1.32 | 2.12e-05 | 5.29e-05 | 1.31 |  |  | list of regions | genes list |
| MA0156.1 | FEV | 5.27% | 3.76% | 1.32 | 3.14e-06 | 8.26e-06 | 1.12 |  |  | list of regions | genes list |
| MA0470.1 | E2F4 | 29.27% | 22.49% | 1.29 | 6.50e-23 | 1.08e-21 | 1.32 |  |  | list of regions | genes list |
| MA0509.1 | Rfx1 | 10.94% | 8.27% | 1.29 | 7.33e-09 | 2.62e-08 | 1.39 |  |  | list of regions | genes list |
| MA0076.2 | ELK4 | 14.66% | 11.29% | 1.27 | 1.81e-10 | 1.13e-09 | 1.58 |  |  | list of regions | genes list |
| MA0527.1 | ZBTB33 | 13.23% | 10.29% | 1.26 | 5.76e-09 | 2.40e-08 | 1.51 |  |  | list of regions | genes list |
| MA0062.2 | GABPA | 14.56% | 11.38% | 1.26 | 1.59e-09 | 7.21e-09 | 1.69 |  |  | list of regions | genes list |
| MA0600.1 | RFX2 | 11.70% | 9.11% | 1.26 | 6.38e-08 | 2.13e-07 | 1.27 |  |  | list of regions | genes list |
| MA0024.2 | E2F1 | 24.70% | 19.53% | 1.25 | 2.36e-15 | 1.97e-14 | 1.29 |  |  | list of regions | genes list |
| MA0059.1 | MYC::MAX | 6.00% | 4.75% | 1.22 | 4.13e-04 | 8.59e-04 | 1.28 |  |  | list of regions | genes list |
| MA0048.1 | NHLH1 | 22.62% | 18.62% | 1.20 | 3.24e-10 | 1.62e-09 | 1.39 |  |  | list of regions | genes list |
| MA0145.2 | Tcfcp2l1 | 26.35% | 22.06% | 1.19 | 2.09e-10 | 1.16e-09 | 1.47 |  |  | list of regions | genes list |
| MA0014.2 | PAX5 | 23.30% | 19.56% | 1.18 | 6.66e-09 | 2.56e-08 | 1.43 |  |  | list of regions | genes list |
| MA0107.1 | RELA | 7.18% | 5.94% | 1.18 | 1.51e-03 | 3.01e-03 | 1.02 |  |  | list of regions | genes list |
| MA0519.1 | Stat5a::Stat5b | 6.18% | 5.11% | 1.18 | 3.15e-03 | 5.83e-03 | 1.13 |  |  | list of regions | genes list |
| MA0146.2 | Zfx | 41.26% | 35.03% | 1.17 | 4.41e-16 | 4.41e-15 | 1.40 |  |  | list of regions | genes list |
| MA0510.1 | RFX5 | 18.72% | 15.87% | 1.17 | 1.82e-06 | 5.06e-06 | 1.31 |  |  | list of regions | genes list |
| MA0101.1 | REL | 7.10% | 6.01% | 1.16 | 5.41e-03 | 9.34e-03 | 1.10 |  |  | list of regions | genes list |
| MA0524.1 | TFAP2C | 37.18% | 33.24% | 1.12 | 1.69e-07 | 4.96e-07 | 1.34 |  |  | list of regions | genes list |
| MA0138.2 | REST | 24.88% | 22.27% | 1.11 | 9.84e-05 | 2.24e-04 | 1.34 |  |  | list of regions | genes list |
| MA0003.2 | TFAP2A | 32.98% | 29.95% | 1.10 | 3.58e-05 | 8.54e-05 | 1.33 |  |  | list of regions | genes list |
| MA0597.1 | THAP1 | 22.89% | 20.86% | 1.09 | 1.86e-03 | 3.58e-03 | 1.32 |  |  | list of regions | genes list |
| MA0139.1 | CTCF | 28.56% | 26.51% | 1.07 | 3.82e-03 | 6.83e-03 | 1.28 |  |  | list of regions | genes list |

Haystack was built by Luca Pinello at Yuan Lab | Contact Author
