# Supplementary material for: Oestrogen receptor β regulates epigenetic patterns at specific genomic loci through interaction with thymine DNA glycosylase
Source: Epigenetics Chromatin. 2016 Feb 16;9:7. doi: 10.1186/s13072-016-0055-7 (PMC4756533; doi:10.1186/s13072-016-0055-7)
Supplement: Supplementary file 6 — 10.1186/s13072-016-0055-7 Links to enriched transcription factor motifs identified using Haystack software. [file 13072_2016_55_MOESM6_ESM.html]

Haystack - Motif enrichment Analysis


Haystack Motif Enrichment Analysis Tool

Target Coordinates: ./hyper.bed (2055)
  
Background Coordinates: RRBS\_background.bed (4110)
  
Motifs database file:/opt/apps/Haystack/motif\_databases/JASPAR\_CORE\_2014\_vertebrates.meme
  
Command used: */opt/apps/Haystack/bin/haystack\_motifs ./hyper.bed mm9 --bed\_bg\_filename RRBS\_background.bed*

| Motif ID | Motif Name | Presence in Target | Presence in BG | Ratio | p-value | q-value | Central Enrichment | Motif Profile | Logo | Regions with Motif | Nearby Genes |
| --- | --- | --- | --- | --- | --- | --- | --- | --- | --- | --- | --- |
| MA0091.1 | TAL1::TCF3 | 6.42% | 4.18% | 1.43 | 1.75e-04 | 2.01e-03 | 1.17 |  |  | list of regions | genes list |
| MA0499.1 | Myod1 | 18.98% | 14.01% | 1.33 | 6.14e-07 | 5.65e-05 | 1.42 |  |  | list of regions | genes list |
| MA0500.1 | Myog | 18.54% | 13.80% | 1.32 | 1.58e-06 | 7.29e-05 | 1.41 |  |  | list of regions | genes list |
| MA0521.1 | Tcf12 | 17.57% | 13.38% | 1.29 | 1.66e-05 | 4.11e-04 | 1.39 |  |  | list of regions | genes list |
| MA0519.1 | Stat5a::Stat5b | 8.37% | 6.33% | 1.28 | 3.57e-03 | 2.23e-02 | 1.10 |  |  | list of regions | genes list |
| MA0144.2 | STAT3 | 10.51% | 8.03% | 1.27 | 1.43e-03 | 1.10e-02 | 1.19 |  |  | list of regions | genes list |
| MA0092.1 | Hand1::Tcfe2a | 7.64% | 5.79% | 1.27 | 5.79e-03 | 3.33e-02 | 1.11 |  |  | list of regions | genes list |
| MA0522.1 | Tcf3 | 19.51% | 15.13% | 1.27 | 1.79e-05 | 4.11e-04 | 1.33 |  |  | list of regions | genes list |
| MA0510.1 | RFX5 | 16.11% | 12.55% | 1.26 | 1.63e-04 | 2.01e-03 | 1.43 |  |  | list of regions | genes list |
| MA0002.2 | RUNX1 | 12.85% | 10.00% | 1.26 | 8.57e-04 | 7.89e-03 | 1.22 |  |  | list of regions | genes list |
| MA0065.2 | PPARG::RXRA | 21.17% | 17.03% | 1.23 | 9.37e-05 | 1.44e-03 | 1.11 |  |  | list of regions | genes list |
| MA0103.2 | ZEB1 | 12.94% | 10.41% | 1.22 | 3.63e-03 | 2.23e-02 | 1.28 |  |  | list of regions | genes list |
| MA0003.2 | TFAP2A | 26.28% | 21.46% | 1.21 | 2.64e-05 | 4.86e-04 | 1.55 |  |  | list of regions | genes list |
| MA0088.1 | znf143 | 17.76% | 14.57% | 1.20 | 1.37e-03 | 1.10e-02 | 1.13 |  |  | list of regions | genes list |
| MA0524.1 | TFAP2C | 27.98% | 24.21% | 1.15 | 1.56e-03 | 1.10e-02 | 1.48 |  |  | list of regions | genes list |

Haystack was built by Luca Pinello at Yuan Lab | Contact Author
