# Supplementary material for: Oestrogen receptor β regulates epigenetic patterns at specific genomic loci through interaction with thymine DNA glycosylase
Source: Epigenetics Chromatin. 2016 Feb 16;9:7. doi: 10.1186/s13072-016-0055-7 (PMC4756533; doi:10.1186/s13072-016-0055-7)
Supplement: Supplementary file 8 — 10.1186/s13072-016-0055-7 Primers used for real-time PCR and Pyrosequencing. MSD: methylation sensitive restriction enzyme digest, pyro: pyrosequencing. [file 13072_2016_55_MOESM8_ESM.pdf]

**Primers used for real-time PCR and Pyrosequencing.** MSD: methylation sensitive restriction enzyme digest, pyro: pyrosequencing.

| Target | PCR primers | Sequencing primer | Method |
|---|---|---|---|
| Hypo1 | for: TACCTAGCGGGTGGACATTC<br>rev: TCCGGGTCTGCAGTAGAGTC | | MSD |
| Hypo2 (*Dyx1c1*) | for: GGCTTCCTAGCGACGTAAAG<br>rev: TTTCCCCACAGAATACGTCAG | | MSD, ChIP |
| Hypo3 | for: AGAGCTGCAAGGCAGATGAT<br>rev: TTTTGCCTCCAAGAATCCAT | | MSD |
| Hypo4 | for: TTCATGGGGAATTAACGCATA<br>rev: AAGGATGACGCACACTCTTTC | | MSD |
| Hypo5 | for: CTCTGAGTGTTTGTCCCTGTG<br>rev: CTACTCCAATTCCAGGGCAT | | MSD |
| Hypo6 | for: TGTGTTCCGTACCACCATTG<br>rev: GGTCTACCCGCACTTTTTCA | | MSD |
| Hypo7 | for: GAGCCCAGAGCCCATACTC<br>rev: ACCCTTTAAGGCAGGGATA | | MSD |
| Hypo8 (*HoxD9*) | for: CCCAGTTTAGCGGACTGATT<br>rev: CTGCAGCCACCATTG | | MSD, ChIP |
| Hypo9 | for: AAAACTTCGGCCAACATGAG<br>rev: TGAGCCTGCAGTTCTCTGTG | | MSD |
| Hypo10 | for: GCCCTCAATCGCAGACACTA<br>rev: GTTTTAACTGCCAGCCATGC | | MSD |
| Hyper1 (*HoxA9*) | for: GTGAGTGTCAAGCGTGGGACAGTCA<br>rev: CTCTCCTTCGCGGGCTTAC | | MSD, ChIP |
| Hyper2 (*HoxA10*) | for: AGCTCGCTAGTCCCTTTCCT<br>rev: GTCGAGCCAGAGGCCAGT | | MSD, ChIP |
| Hyper3 | for: ACTGGCCTCTGGCTCGAC<br>rev: CACCACCCACTCTGGTTTG | | MSD |
| Hyper4 (*Tnfaip2*) | for: GCCCCCAACACTGGACTC<br>rev: GGGCCCTCCTAAAATAGTCG | | MSD, ChIP |
| Hyper5 | for: TGCTGTCTGTCATTTGCTCTG<br>rev: CTCCAGGCCTCTGAGGAAG | | MSD |
| Hyper6 (*Pitx1*) | for: TTGTCTGCAGCAGGTAGGAA<br>rev: CTGGATGGAGGGAGGGAAG | | MSD, ChIP |
| Hyper7 | for: CTATTCCAGTCGGAGCCATC<br>rev: GGAATGGGCCTGAGAAACA | | MSD |
| Hyper8 | for: GACCGTCCCTGCAGAGTTAT<br>rev: CGGGGCTGCAGTTAATAAAG | | MSD |
| *Dyx1c1* | for: TTGTAATATTTAATATAGTGGGAGAT<br>for_nest: AGGGTAGGAAGTGATGGTAATT<br>rev: AAAAAATTAACCTTCAAATAACTTTCC (biot) | Seq1: GGAAGTGATGGTAATTT<br>Seq2: AGTGGTTGTGTAGTT | Pyro |
| *HoxA9* | for: TAGTTAGGGATAAAGTGTGAGTGTTAAG<br>for_nested: GTGAGTGTTAAGAGTGGGATAGTTAT<br>rev: ACTACAATATATCATCACCACCACC (biot) | GTTTAATGTTATAAGGT | Pyro |
| *Pitx1* | for: TGTTGGTTTGTGAAGTGAGTG<br>rev: ATTCCCAAAACCCCTAAATAAAA (biot) | TTTGTTTGTAGTAGGTAGGAATG | Pyro |
| *Esrrg* | for: TATTAAGAAAATAGGGAAGGTATTATGA | GGAAGGTATTATGAAGTTTTGGGTTATAGG | Pyro |

| | | | |
|---|---|---|---|
| | rev: CCTCCTTAAACTAAACTCCC (biot) | | |
| *HoxA11* | for: AGAAGGGGTTTAAGTAGT<br>rev: CCACCCACCTTATCCTCAA (biot) | GGAGGAGAAGGAGAGA | Pyro |
| *Trim2* | for: TATAGTAGATTTTTTAAAAGGGGGT<br>rev: CTCAAACCTACCAAAAAACCTTATA<br>rev_nest:<br>AAAAAAAACAACATAAAACTAAACC<br>(biot) | GTTGTGAATAGAGGTATTGG<br>ATAAGTGA | Pyro |
| *Fstl4* | for: GTGAAGTTAGAGGGGATAATAGTGA<br>for_nested:<br>GGGTTTTTTTGTTTTGTAGGTTTAG<br>rev:<br>TTTCCTAAACAAATTAATCCCTTAATAT<br>C (biot) | AGGTTTAGATATTTTTTTTAT | Pyro |
| *Dyx1c1* | for: TGCGCGATGCTGACGTATT<br>rev: AGAAAGGCTATCCCACAGAACT | | RT-qPCR |
| *HoxD9* | for: GCCACTACGGGATTAAGCCTG<br>REV: GCGGAGCACTCAGTCCTTT | | RT-qPCR |
| *HoxA9* | for: GGCCTTATGGCATTAAACCTGA<br>rev: ACAAAGTGTGAGTGTCAAGCG | | RT-qPCR |
| *HoxA10* | for: GGAAGCATGGACATTCAGGT<br>rev: CCAGGCAAGCAAGACCTTAG | | RT-qPCR |
| *Tnfaip2* | for: AGGAGGAGTCTGCGAAGAAGA<br>rev: GGCAGTGGACCATCTAACTCG | | RT-qPCR |
| *Pitx1* | for: ATCGTCCGACGCTGATCTG<br>rev: GCTTGTGAAGTGAGTGCGTT | | RT-qPCR |
| *β-actin* | for: CGTCGACAACGGCTCCGGCATG<br>rev: CCACCATCACACCCTGGTGCCTAGG | | RT-<br>qPCRs |
