# Supplementary material for: Oestrogen receptor β regulates epigenetic patterns at specific genomic loci through interaction with thymine DNA glycosylase
Source: Epigenetics Chromatin. 2016 Feb 16;9:7. doi: 10.1186/s13072-016-0055-7 (PMC4756533; doi:10.1186/s13072-016-0055-7)
Supplement: Supplementary file 9 — 10.1186/s13072-016-0055-7 Antibodies used for western blotting and ChIP. [file 13072_2016_55_MOESM9_ESM.pdf]

Antibodies used for western blotting and ChIP

**Name**	**Company/Reference**	**Method**
α-TDG 141 Salasso	P. Schär [1]	Western blot/ChIP
α-ERβ LBD IgG	M. Warner [2]	Western blot/ChIP
α-GST	GE Healthcare	Western blot
α-Hsp90 F-8	Santa Cruz	Western blot

1. Hardeland U, Steinacher R, Jiricny J, Schar P. Modification of the human thymine-DNA glycosylase by ubiquitin-like proteins facilitates enzymatic turnover. The EMBO journal 2002; 21:1456-64.
2. Saji S, Jensen EV, Nilsson S, Rylander T, Warner M, Gustafsson JA. Estrogen receptors alpha and beta in the rodent mammary gland. Proceedings of the National Academy of Sciences of the United States of America 2000; 97:337-42.
